# Clinical relevance of "withdrawal therapy" as a form of hormonal manipulation for breast cancer

**DOI:** 10.1186/1477-7819-9-101

**Published:** 2011-09-09

**Authors:** Amit Agrawal, John FR Robertson, KL Cheung

**Affiliations:** 1 Division of Breast Surgery, GEM School, University of Nottingham, Royal Derby Hospital, Derby DE22 3NE, UK

## Abstract

**Background:**

It has been shown in in-vitro experiments that "withdrawal" of tamoxifen inhibits growth of tumor cells. However, evidence is scarce when this is extrapolated into clinical context. We report our experience to verify the clinical relevance of "withdrawal therapy".

**Methods:**

Breast cancer patients since 1998 who fulfilled the following criteria were selected from the departmental database and the case-notes were retrospectively reviewed: (1) estrogen receptor positive, operable primary breast cancer in elderly (age > 70 years), locally advanced or metastatic breast cancer; (2) disease deemed suitable for treatment by hormonal manipulation; (3) disease assessable by UICC criteria; (4) received "withdrawal" from a prior endocrine agent as a form of therapy; (5) on "withdrawal therapy" for ≥ 6 months unless they progressed prior.

**Results:**

Seventeen patients with median age of 84.3 (53.7-92.5) had "withdrawal therapy" as second to tenth line of treatment following prior endocrine therapy using tamoxifen (n = 10), an aromatase inhibitor (n = 5), megestrol acetate (n = 1) or fulvestrant (n = 1). Ten patients (58.8%) had clinical benefit (CB) (complete response/partial response/stable disease ≥ 6 months) with a median duration of Clinical Benefit (DoCB) of 10+ (7-27) months. Two patients remain on "withdrawal therapy" at the time of analysis.

**Conclusion:**

"Withdrawal therapy" appears to produce sustained CB in a significant proportion of patients. This applies not only to "withdrawal" from tamoxifen, but also from other categories of endocrine agents. "Withdrawal" from endocrine therapy is, therefore, a viable intercalating option between endocrine agents to minimise resistance and provide additional line of therapy. It should be considered as part of the sequencing of endocrine therapy.

## Background

Estrogen receptor (ER) positive breast cancers after a period of response to anti-estrogens develop resistance and clinically the disease progresses. Besides the predominant role of alternative signalling pathways, domination of partial agonistic activity of tamoxifen over its antagonist activity has been implicated for acquired resistance [1]. Regression of tumor on cessation of tamoxifen therapy and the resultant clinical benefit (CB) have been reported in several case-reports and series [2-6]. In-vitro experiments have also shown that "withdrawal" of tamoxifen inhibits growth of tumor cells [7]. We report clinical relevance of "withdrawal therapy" from tamoxifen and other hormonal agents in patients heavily pre-treated with endocrine therapy.

## Methods

Case-notes of the breast cancer patients treated in the Nottingham breast unit since 1998 fulfilling the following criteria were studied retrospectively:

• ER positive invasive breast carcinoma proven by histology (Standard Immuno-histochemically estimated H score ≥ 50 accepted as ER +) [8]

• Primary operable cancer in elderly (age > 70 years) (who were frail or refused to undergo surgery), locally advanced or metastatic

• Disease deemed suitable for further hormonal manipulation

• Disease progressed on a hormonal agent and thus suitable for "withdrawal" from an endocrine agent as a therapeutic option (as opposed to a palliative option)

• Assessable lesions were deemed to have shown CB when they either had objective response in the form of complete response (CR) or partial response (PR); or had stable disease (SD) for ≥ 6 months in accordance with UICC criteria [9,10]

• Metastatic lesions were assessed radiologically (CT scan/X-rays/bone scan) every 3 months as routine protocol in the unit

• On "withdrawal therapy" for at least 6 months unless disease progressed prior

Duration of CB (DoCB) is the duration of therapy in months only in patients who have derived CB and including patient still on treatment. Duration of treatment (DoT) is the duration of therapy in months of all patients (regardless of the type of response) and including patient still on treatment

## Results

Seventeen patients with either locally advanced primary (n = 3) or metastatic (n = 14) breast cancer had "withdrawal" treatment as 2nd to 10th line of treatment. Patient and tumor characteristics are shown in Table 1. Two patients were still on follow-up at analysis. The results from "withdrawal" from different agents are shown in the Table 2. Drugs prior to withdrawal were mostly several lines of endocrine agents (tamoxifen, aromatase inhibitors, AIs- letrozole and exemestane, fulvestrant, megestrol acetate) but some had radio and chemotherapy as necessary. However, in current study, only patients in whom the preceding therapy was an endocrine agent were considered for withdrawal (as mentioned in Table 2).

**Table 1:** Patient and Tumour characteristics

Median age =	84.3 (53.7-92.5) years
**Histopathology**	Invasive adenocarcinoma = 12 Mixed Tubular + Cribriform = 1 Not available = 4
**Median ER (estrogen receptor) H score**	185 (IQR, 97.5-222)
**Median Disease Free Interval (DFI)**	84 (IQR, 19-180) months
**Metastatic Sites**	Bone = 6 Pleura/Effusion = 4 Lung = 2 Liver = 5 Supraclavicular/mediastinal Lymph node = 2
**Median lines of therapy prior to withdrawal**	5 (range 1-9)
**Median TTP (time to progression) on endocrine agent prior to withdrawal**	6 (IQR, 3-17) months

**Table 2:** Disease distribution and Clinical results

Patients (n)	Disease type	CB = n (%)	DoCB in months	DoT in months
**All (n = 17)**	14 MBC 3 LAPC	10 (58.8%); 1PR,9SD (8 MBC & 2 LAPC)	10+ (7-27)	9+ (1-27)
**Tamoxifen (n = 10)**	9 MBC 1 LAPC	6 (60%); 1 PR, 5 SD (6 MBC)	10.5+ (7-27)	8.5+ (1-27)
**Rest (n = 7)** **1M,1F,4E,1L**	5 MBC 2 LAPC	4 (57.1%); 4 SD (2 MBC & 2 LAPC)	9.5+ (9-23)	9+ (2-23)

**M **= Megestrol acetate; **F **= Fulvestrant; **E **= Exemestane; **L **= Letrozole; **MBC **= Metastatic Breast Cancer; **LAPC **= Locally Advanced Primary Breast Cancer; **PR **= Partial Response; **SD **= Stable Disease; **DoCB **= Duration of Treatment in patients with Clinical Benefit; **DoT **= Duration of Treatment irrespective of response

## Discussion

Tamoxifen may continue to provide antagonistic activity on ER nuclear signalling activity but may act as an agonist on the ER membrane signalling activity which could explain the loss of continuing CB to some patients on long-term tamoxifen therapy [1]. There is significant and sustained CB (60%) on "withdrawal" from tamoxifen even in heavily pre-treated patients in our study. Previous studies have explained development of resistance to tamoxifen itself due to clonal selection of breast cancer cells that grow in the presence of tamoxifen [4,6]. An in vitro study of cells derived from tumors of post-menopausal patients which progressed on tamoxifen showed growth enhanced by addition of tamoxifen [11] suggesting domination of agonistic activity on long-term tamoxifen therapy.

In an in-vivo study [12], athymic mice were transplanted with ER positive tumor cells and then exposed to tamoxifen or placebo. The tumors regressed initially over 4 months but started to grow towards the end of the study (8 months) on long-term exposure to tamoxifen and placebo. Tumors from both groups were re-transplanted into athymic mice. Tumors which grew in the presence of tamoxifen grew either on exposure to estrogen or on exposure to further tamoxifen. In clinical setting, therefore, cessation of further growth would be expected on withdrawal of tamoxifen therapy at development of resistance to tamoxifen. Our case-series and previously reported case series confirm this finding in clinical setting.

The lack of response in the remaining patients on withdrawal of tamoxifen could partly be explained by the growth promoting action of natural estrogen in the body as seen in the above in-vivo study [12]. Therefore, if "withdrawal therapy" from tamoxifen does not provide any clinically beneficial response, an AI or fulvestrant may be the subsequent endocrine agents of choice to negate the effects of persisting estrogen. This tactic of manipulating hormonal environment of the tumor provides a viable intercalating option between endocrine agents without possible side-effects.

In our study, as seen in table 2, there were a group of patients who were on endocrine agents other than tamoxifen (megestrol acetate, aromatase inhibitors, and fulvestrant). Therapy in these patients was withdrawn either due to side-effects or patient unfitness/refusal. No further endocrine therapy was instituted as they already had multiple lines of other therapies. On routine clinical follow-up, incidental responses were seen in these patients. It is difficult to explain incidental responses to withdrawal from categories of endocrine agents other than tamoxifen or an AI. It could be due to paradoxical action of natural estrogens rebounding after "withdrawal" of anti-estrogens without agonist activity [1,13]. In an in-vitro study in MCF-7 cells by Masamura et al [14] long-term estrogen deprivation (akin to usage of anti-estrogens in the form of AIs clinically) led these cells to develop estrogen hypersensitivity. In a long-term estrogen deprived aromatase resistant breast cancer cell model (MCF-7:5C), Osipo et al [7] demonstrated apoptosis at a concentration of 10^-11 ^M/L or more of estradiol. This is again possible due to activation of cross-talk mechanisms and domination of mitogen-activated protein kinase or growth factors [1].

The above in-vitro concept is being explored further in clinical settings. There is ongoing recruitment to a phase III trial (SOLE trial, Study Of Letrozole Extension trial) wherein node positive early stage patients who have completed 4-6 years of prior adjuvant endocrine therapy (a SERM/AI/both) are randomised to receive either 5 years of daily letrozole or receive intermittent letrozole regime (daily for first 9 months of 1^st ^4 years followed by 12 months in year 5)[15]. The rationale for this study is that long-term estrogen deprivation by an AI reduces the sensitivity of the tumour cells to therapy and interruptions to therapy allows resurgence of estrogenic stimulation leading to restoration of sensitivity to letrozole on its reintroduction.

In locally advanced and advanced breast cancer, ideally, inhibitors of the alternative pathways would be the subsequent logical therapy following resistance to other endocrine agents. However, if the patient is unfit to receive any further anti-estrogen therapy then it is very likely that they would not be suitable for further growth factor inhibitors or indeed chemotherapeutic agents. In this circumstance, withdrawal of therapy may be the only feasible option or alternating regime of endocrine therapy and withdrawal therapy as is being tested in the adjuvant setting in the SOLE trial.

## Conclusions

Our data therefore provides some clinical evidence and emphasises the relevance of withdrawal therapy. The concept is being tested in large randomised trials in adjuvant settings. However, larger datasets and results of ongoing adjuvant trials are needed to provide confirmatory evidence for or against the concept and feasibility of withdrawal therapy in locally advanced and metastatic breast cancer.

## Conflict of interests

The authors declare that they have no competing interests.

## Authors' contributions

KLC conceived this study. Patients were under care of JFR and KLC. AA collected data, performed analysis, drafted, revised and finalised the manuscript. KLC and JFR revised and approved of the contents of the manuscript. All authors read and approved the final manuscript.
